# Modelling the interplay of SARS-CoV-2 variants in the United Kingdom

**DOI:** 10.1038/s41598-022-16147-w

**Published:** 2022-07-20

**Authors:** N. L. Barreiro, T. Govezensky, C. I. Ventura, M. Núñez, P. G. Bolcatto, R. A. Barrio

**Affiliations:** 1 Instituto de Investigaciones Científicas y Técnicas para la Defensa (CITEDEF), 1603 Buenos Aires, Argentina; 2 Instituto de Investigaciones Biomédicas, Universidad Nacional Autónoma de México, 04510 Mexico, Mexico; 3 (CONICET) Centro Atómico Bariloche-CNEA, Universidad Nacional de Río Negro, 8400 Bariloche, Argentina; 4 Consejo Nacional de Investigaciones Científicas y Técnicas (CONICET), Buenos Aires, Argentina; 5 Departamento Materiales Nucleares, Centro Atómico Bariloche, Comisión Nacional de Energía Atómica (CNEA), Bariloche, Argentina; 6 INIBIOMA, Universidad Nacional del Comahue, Bariloche, Argentina; 7 Instituto de Matemática Aplicada del Litoral (IMAL, CONICET/UNL), FHUC, Santa Fe, 3000 Argentina; 8 Instituto de Física, Universidad Nacional Autónoma de México, Apartado Postal 20-365, 04510 Mexico, Mexico

**Keywords:** Epidemiology, Statistical physics, thermodynamics and nonlinear dynamics

## Abstract

Many COVID-19 vaccines are proving to be highly effective to prevent severe disease and to diminish infections. Their uneven geographical distribution favors the appearance of new variants of concern, as the highly transmissible Delta variant, affecting particularly non-vaccinated people. It is important to device reliable models to analyze the spread of the different variants. A key factor is to consider the effects of vaccination as well as other measures used to contain the pandemic like social behaviour. The stochastic geographical model presented here, fulfills these requirements. It is based on an extended compartmental model that includes various strains and vaccination strategies, allowing to study the emergence and dynamics of the new COVID-19 variants. The model conveniently separates the parameters related to the disease from the ones related to social behavior and mobility restrictions. We applied the model to the United Kingdom by using available data to fit the recurrence of the currently prevalent variants. Our computer simulations allow to describe the appearance of periodic waves and the features that determine the prevalence of certain variants. They also provide useful predictions to help planning future vaccination boosters. We stress that the model could be applied to any other country of interest.

## Introduction

Towards the end of 2021 the SARS-CoV-2 virus pandemic has infected more than 500 million people worldwide, with more than 6 million global deaths^1^. Global vaccination has progressed, though at very different rates throughout the world and in the context of a vaccine shortage. While half of the world population has started vaccination schemes, the distribution is highly heterogeneous. In many countries, mainly those of low income, the percentage of the population with immunity processes in progress is lower than 5%^2^. This feature favours the virus spread, and its genome replication originates mutations that lead to new virus variants^3^.

Several hundred thousand new variants have been identified in the SARS-CoV-2 genome^4^. Most of them do not affect the transmissibility of the virus because they do not alter the “spike” protein shape. In spite of this fact, the large amount of infections gave rise to some clinically relevant variants (variants of concern), that are transmitted more easily and rapidly than the original ones^5,6^ and that can even be more lethal^7^. The most widely spread strains include the ones first detected in: the United Kingdom (Alpha or B.1.1.7), South Africa (Beta or B.1.351), Brazil (Gamma or P.1), and India (Delta or B.1.617.2)^8^. The emergence of new COVID-19 variants raises doubts about the effectiveness of vaccination campaigns^9^. Furthermore, these strains allowed the appearance of fast reinfections^10^ and co-infections^11^. The available information suggests that new variants could draw out the pandemic if measures are not taken^12^. Further research is needed to understand the impact of their spread. For instance, Ramos et al.^13^ studied the impact of the introduction of a new variant of the virus on a territory, including vaccination campaigns, for the case or Italy. Another recent work by Okabe and Shudo^14^ focused on the spread of a new variant on top of the original strain, using two contact network topologies, observing no relevant differences on the effect of the new variant. There is also a study based on Ontario^15^ considering the interplay of three different strains, and vaccination, without taking into account the spatial heterogeneity. Accordingly, we propose the development of a stochastic geographic expansion model, that includes several different strains, to study the emergence and dynamics of these new variants. The model is based on a previously used discrete mathematical map that starts from an extended SEIRS model^16–19^. The model is applied to the case of the UK by fitting the actual properties of each strain with the available data up to the appearance of the Delta variant. Vaccination effects are also taken into account in order to study its effectiveness under scenarios with different strains.

## Model

**Figure 1:**
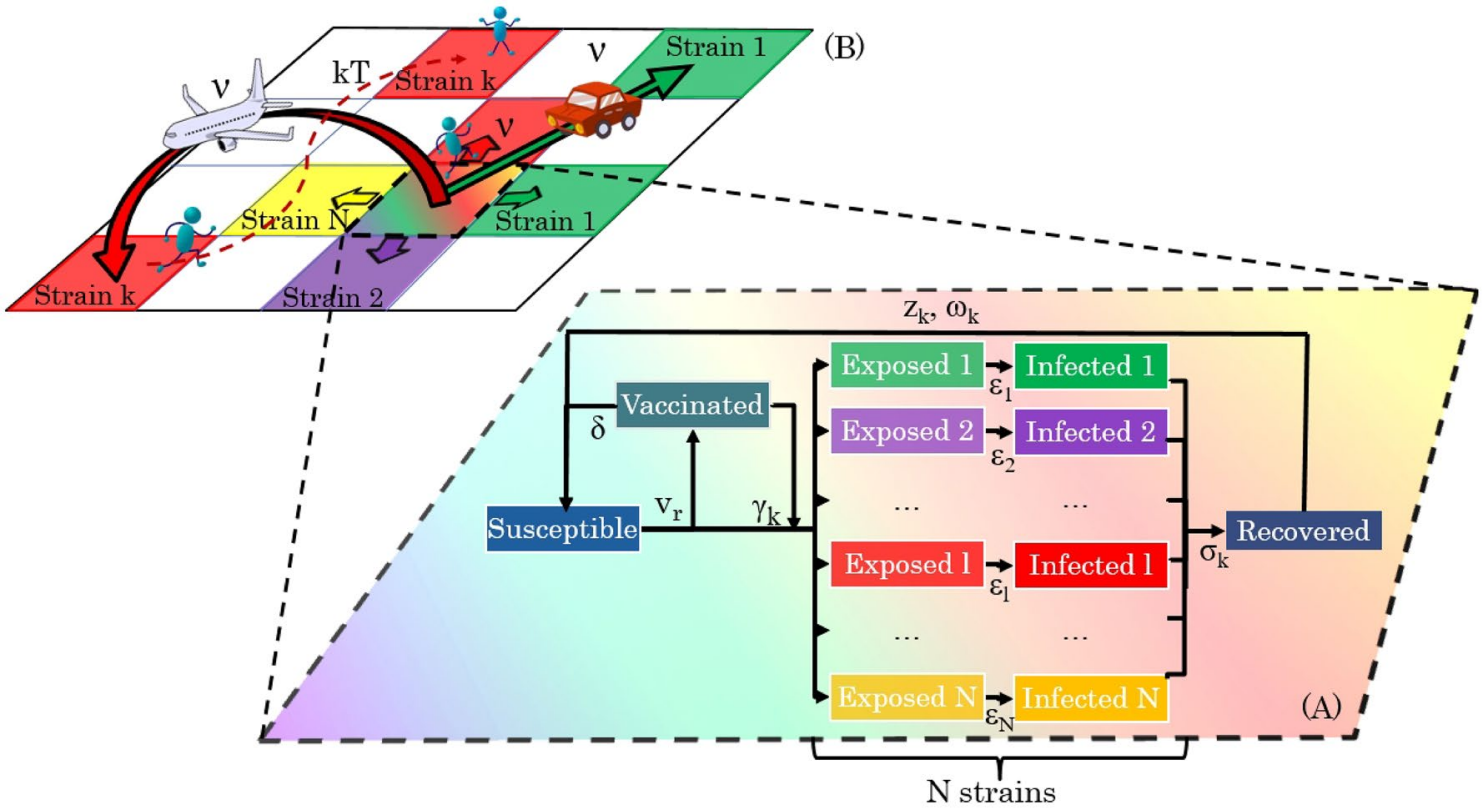
Diagram showing the geo-stochastic model scheme. (**A**) Represents the local dynamics of a SEIRS-V model. The compartments taken into account are: Susceptible, Recovered, Vaccinated, Exposed and Infected with different strains Vaccinated people can get infected with a certain variant *k* with a probability $\gamma _k$. The exposed, infectious and immune periods for the *k*th strain are $\epsilon _k$, $\sigma _k$ and $\omega _k$, respectively. The variables $v_r$ and $\delta$ stand for the vaccination rate and the immunity period conferred by the vaccine. (**B**) The global dynamics on a geographical area, divided into a grid of cells, is followed by placing a SEIRS-V model on each one and allowing contagions between them. Three mobility processes are considered: movement to neighbor and far cells, and thermal noise. (Diagram was made by N.L. Barreiro using standard free software).

The model used in this work is an extension of the one presented by Barreiro et al.^19^ that includes the emergence of new virus variants at different times. The map of the country being studied is divided into a two-dimensional grid in which the dynamics occurs. We use a modified SEIRS-V model to simulate the local dynamics (see Fig. 1A) of infection spreading, taking place in each cell (*i*, *j*) of an area of a few $km^2$, with average population density $\rho (i,j)$. Global dynamics (Fig. 1B), comprising the entire country, considers geographical spread to neighboring cells and along terrestrial roads or aerial routes. Governmental non-pharmaceutical interventions, and social behavior are reflected in the mobility parameters used (*v*).

### Description of the local dynamics

 The compartmental model includes: susceptible (*S*), exposed to the different variants present in the location, yet not infectious ($E_k$, $k=1$,...,*N*), after $\epsilon _k$ days people become infectious and can transmit the variant with which they got infected ($I_k$, $k=1$,...,*N*), they stay in this compartment $\sigma _k$ days and then recover (*R*). Acquired immunity lasts for $\omega _k$ days and then, a proportion $z_k$ of people who survived, become susceptible again (*S*). At a specific time, vaccination designed to protect from the initial variant started, being applied at a rate $v_r$; vaccinated people (*V*) stay immune for $\delta$ days but they may be infected with variant *k*, with a probability $\gamma _k$ (Fig. 1A).

### Description of the global dynamics

 Geographical spread (Fig. 1B) is simulated by using mobility parameters: $\nu _n$ for movement between neighbor cells, and $\nu _a$ for long distance trips by airplane, car, or train. We consider that flows between large cities are greater than flows between small ones, therefore, $\nu _a$ is weighted by $\rho (i,j)\rho (m,n)$ i.e. by the origin and destination population densities. All mobility parameters $0<\nu <1$, interpreted as the probability of traveling from one cell, in which $I_k(i,j,t) \ge \eta$, to another cell, are modeled by a Metropolis Monte-Carlo algorithm (for details see Supplementary Information). When infection is allowed on cell (*m*, *n*), one sets $S(m,n,t)=1-\eta$ and $I_k(m,n,t)=\eta$.

Furthermore, there is the possibility of outbreaks in remote and isolated places, because people sporadically travel to unexpected places, regardless of their population. These random movements may be considered as “kinetic energy” *kT* of the system. In this case we use a Monte-Carlo procedure comparing a random number with the quantity $exp(-kT)$.

Interaction between variants can be included in our model, but until now, very few cases of co-infection with two COVID-19 variants have been reported, so we decided not to include possible interactions in the present work.

As in any model depicting a complex system, it is important to consider its limitations. In this case, one limitation is that people become part of the vaccinated compartment only after receiving the shots recommended by pharmaceutics and having developed immunity. Thus, we are not considering the partial immunity acquired while vaccination is in progress. However, the model could be adapted to include more details on individual dose administration if needed. Another important limitation is that predictions depend on the available information about the mobility parameters. Those parameters depend on population compliance with government interventions and recommendations. Any change in people’s behaviour may swiftly change mobility parameters and consequently modify the virus spreading rate. Finally, most epidemiological parameters, as the periods of natural immunity $\omega$, infection $\sigma$ and vaccine immunity $\delta$ could change depending on the strain. Due to the lack of reliable and complete data about these quantities and for definiteness we used the same values for all the virus variants. This could also limit the prediction capacity of the model.

## Results

**Figure 2:**
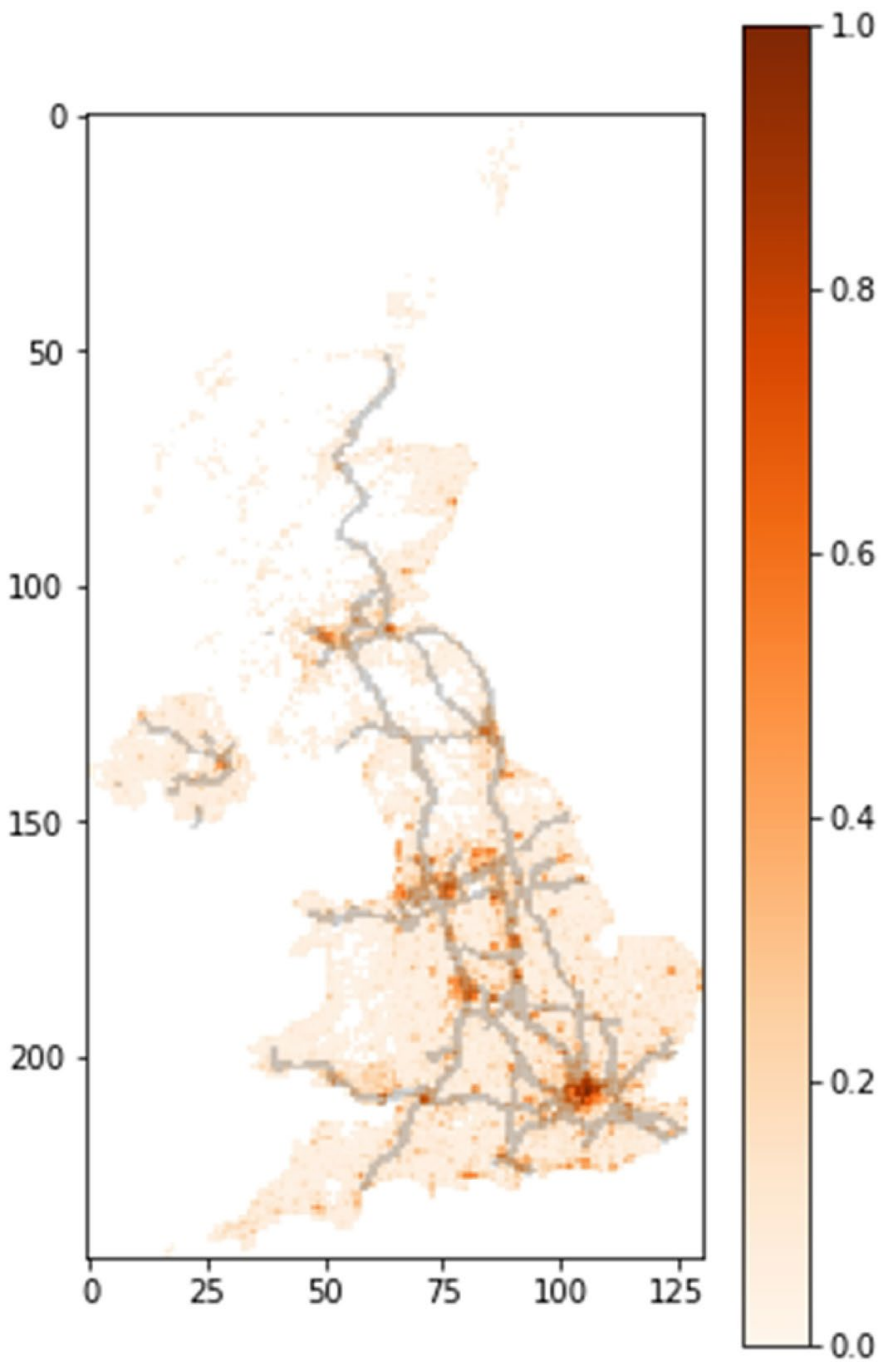
UK normalized population density and main routes map. The map is divided in a grid of squares of 25 km$^2$. The map was generated using custom code^21^.

The model was applied to the case of the United Kingdom (UK). As stated above, to apply the model to a specific country, data about its population density are required. The information to generate the density map was extracted from the “Global High Resolution Population Denominators Project”^20^. The main routes were obtained from Google Maps. In order to compute the geo-stochastic spreading of the virus, the map was divided into a grid of squares, with an area of 25 km$^2$ (see Fig. 2).

The dynamics of different strains of the virus is very well documented in the UK, consequently the available information suffices to fit the parameters of the model for the different variants. For simplicity, only the most abundant strains over time were considered in the adjustment. These are: the EU1 strain (B. 1.177 originated in Europe), the Alpha (B.1.1.7) and the Delta (B.1.617.2). The rest of the variants, less abundant and of less interest, are grouped together under the name “other strains”. Information about the variants was obtained from CoVariants^22^ enabled by data from GISAID^23^. We used the same values of the time delay parameters $\sigma _k$, $\omega _k$ and $z_k$ for all the variants, since, despite this simplification, the model produces very good results. The upper panel of Table 1 shows the values of the parameters used in the calculations.

**Table 1:** Model parameters.

Parameter	Meaning	Value		
$\epsilon _k$	Period before being infectious	1 day $^{(a)}$ $^{(b)}$		
$\sigma _k$	Infectious period	14 days $^{(b)}$		
$\omega _k$	Immunity period	140 days $^{(b)}$		
$z_k$	Survival parameter	0.99		
$\delta$	Vaccination immunity period	180/360 days		
$\eta$	Threshold to start an infection focus	0.00005 $^{(c)}$		

Parameter	Other	EU1	Alpha	Delta
$\gamma _k$	${0}^{*}$	${0}^{*}$	0.01	0.1 / 0.3
$\beta _k$	0.91	1.1	2.3	4.5

The upper panel includes epidemiological parameters common to all strains. The lower panel shows the parameters that differ among strains.

${}^{a}$This value was set to 0 for the Delta variant in order to get a better fit.

${}^{b}$Values fitted and used with this model in previous works^17–19^.

${}^{c}$Corresponds to start of an infection focus with 1 infected persn in an averagely populated area.

${}^{*}$ Values left as zero for simplicity, considering that when massive immunization began, Alpha was the dominant variant.

The vaccination rate $v_r$ was fitted over time in order to adjust the model to the real immunization data (delayed 14 days to ensure immunity development). Data on vaccination and daily cases were obtained from “Our World in Data”^2,24^ and Johns Hopkins University^1^. 66% of the population was fully vaccinated by the end of September. Taking into account the willingness to be vaccinated^2,24^ in the United Kingdom, however, it is not expected that a large population group will be vaccinated after that. For simplicity, in the model we consider that 70% of the population will be immunized.

We used the stringency index^25^ (a measure of the severity of government policies during the pandemic) as a guide to fit mobility $\nu$ over time. In order to reduce the amount of fitting parameters we employed the same value of $\nu$ for both neighbor and far mobility (the latter was weighted by the origin and destination densities). Mobility was adjusted up to August 15, 2021, leaving the same value from then on. Any change in government policies, increasing or decreasing restrictions, could produce a modification of the results. *kT* was left on a low level (0.1) since, from the beginning of the pandemic, there have been multiple measures to reduce mobility (internal restrictions and bans or quarantine for travellers entering the UK from high-risk regions)^2,25^. The parameter $\beta _k$, which reflects how contagious a strain is, was changed to fit the actual curves for each variant. As we mentioned before, new variants could be more contagious^5,6^ and suppress previous ones. In this sense, $\beta _k$ determines how variants will compete and eventually become the dominant strain over time. The lower panel on Table 1 shows the fitted $\beta _k$ values for each strain. At the same time, some vaccines have exhibited a lower effectiveness against some variants^26^. To take this into account we used the $\gamma _k$ parameter. Given a *k*th strain, the higher $\gamma _k$, the more likely that a vaccinated person will get infected by it. Some parameters such as the period of immunity conferred by the vaccine and the efficiency for some variants still need further study. We emphasize here that the immunity period that the vaccines provide, together with the inherent efficiency of the vaccines are the important factors to be considered. Therefore, we decided to propose three possible different scenarios:

- Short vaccine immunity period ($\delta = 180$ days) and relatively high vaccine efficiency against delta variant ($\gamma _{delta} = 0.1$)
- Long vaccine immunity period ($\delta = 360$ days) and low vaccine efficiency against delta variant ($\gamma _{delta} = 0.3$)
- Long vaccine immunity period ($\delta = 360$ days) and relatively high vaccine efficiency against delta variant ($\gamma _{delta} = 0.1$)

**Figure 3:**
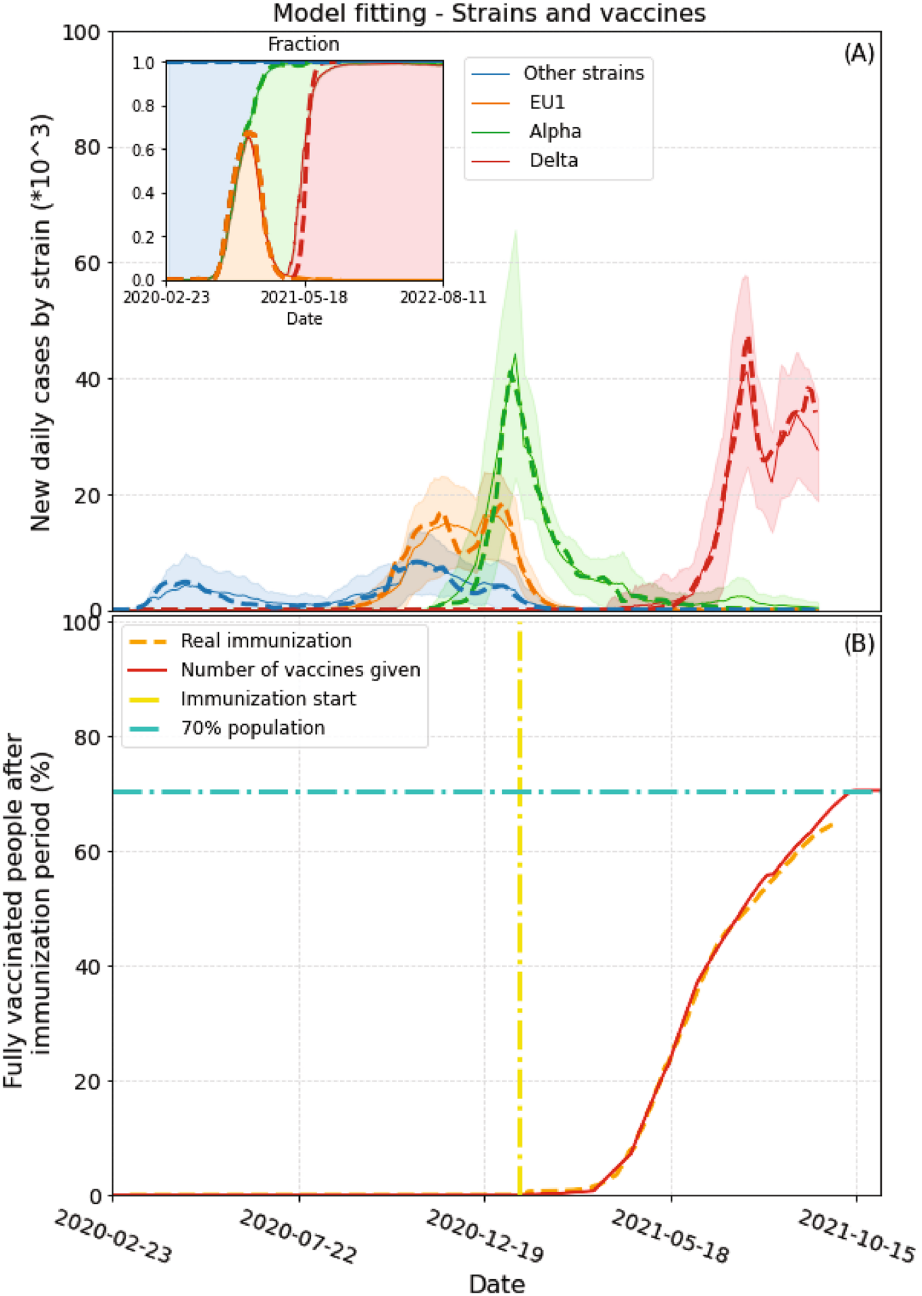
Model fitting to strain and vaccination data. (**A**) Daily cases differentiated by strain. Dashed lines represent actual cases of each strain (scaled). Solid lines and shaded areas represent the average and standard deviations obtained from 100 runs of the model, respectively. The inset shows the fraction of cases for each variant for a longer period of time. The Delta variant is expected to prevail if no new more contagious variants appear. (**B**) Immunization over time. Yellow dash-dot line signals the day immunization started. Red solid line represents the percentage of fully immunized people in the model in time. The dashed orange line depicts the actual two-dose vaccination data (with 14 days delay, which is the average time needed to acquire full immunity after the second dose).

When computing the dynamics of the model, we adjusted $\beta _k$ and $v_r$ to fit the new cases of each variant and the real vaccination rate, respectively. Figure 3A shows the fitting for each variant until September 10. Adjusted values of $\beta _k$ are shown in Table 1. The inset in the figure shows the fraction of infections with each variant. In order to fit the fast case growth of the delta variant (which cannot be explained only in terms of mobility changes) we had to use a very high $\beta _{delta}$ value and a lower value of $\epsilon _{delta}$ (0) (see Table 1). In order to emphasize the evolution of the Delta variant, in the inset we show a longer period of time. From Fig. 3A it is clear that at the current transmission rate this strain will become dominant and, unless a new, more contagious, variant appears, it will prevail in the near future. Figure 3B shows immunization as a function of time. In this case the vaccination rate was changed on the model to fit the actual data with a delay of 14 days. This delay was added to ensure that fully vaccinated people reach actual immunization. The dashed orange curve represent official data while the red solid line corresponds to the model immunization progress. As mentioned before, vaccination stops when 70% population is reached

**Figure 4:**
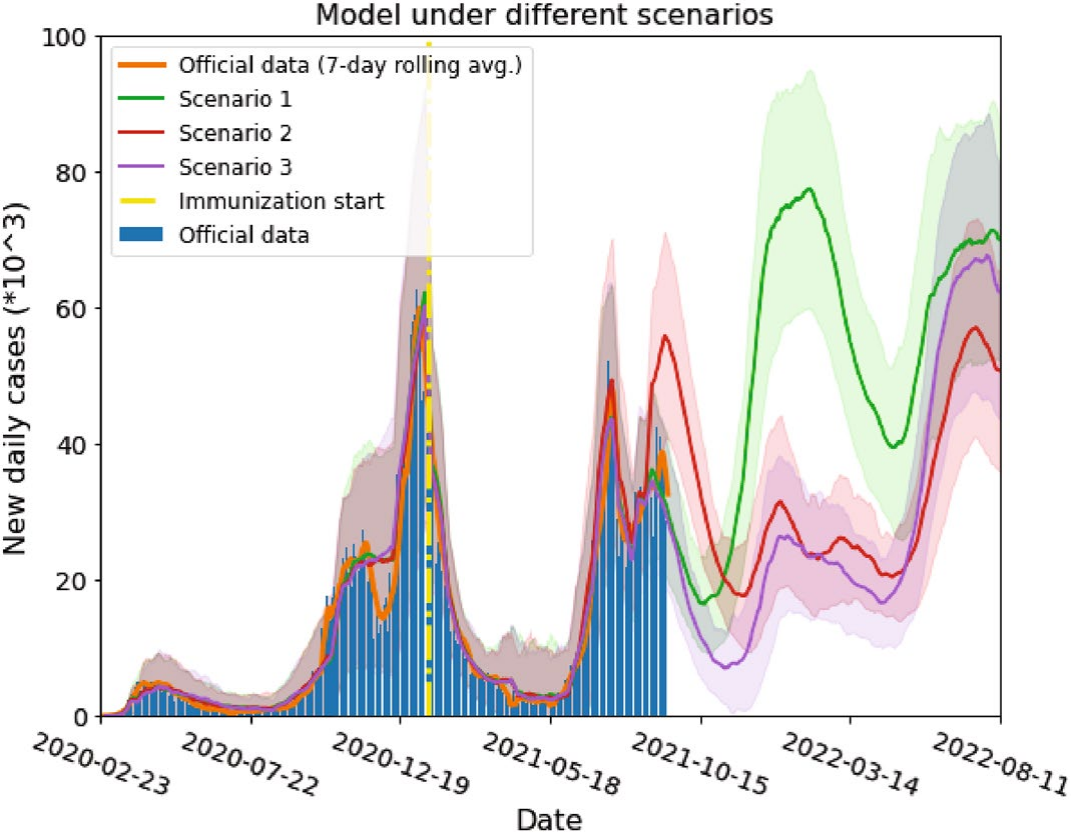
Model results for three different scenarios. Red, green and violet solid lines are the model simulation results under scenarios 1, 2 and 3 (see text), respectively. Shaded areas on each color represent the standard deviation from 100 model runs for each scenario. Yellow dash-dot vertical line indicates the day when immunization started. Blue bars and solid orange lines are the actual daily cases and their 7 day rolling average, respectively. In scenario 1, a short vaccination immunity period implies a growth of the daily cases in the near future, if current mobility is sustained. Under scenario 2, people are expected to be immune for a longer time and breakthrough infections will act as antibodies boosters, prolonging the defence against the pandemic. In scenario 3 most people will be immune in the near future, lowering the number of cases, and a new wave would appear when vaccine immunity ends.

Figure 4 shows the three scenarios mentioned above. The orange solid line represents a 7-day moving average of the new daily cases which are depicted by the blue bars. The start of the vaccination in the model is indicated with a yellow dash-dot line. The model evolution under the first scenario is represented by the green solid line. With a high efficiency vaccine, a low amount of breakthrough infections (infected while fully vaccinated) is expected in the short term, at the current mobility rate. On the other hand, a short immunity period (only 180 days) leads to a massive COVID-19 outbreak for the beginning of 2022, if no restriction measures are imposed in the future.

The model applied to the second scenario is shown in red. As we mentioned before, the model was conceived with the assumption that if a person presents a breakthrough infection, his/her immune system is boosted and therefore he/she will remain immune $\delta$ additional days after infection. This assumption is sensible, given the abundant evidence of increased antibody levels when a previously infected person is vaccinated^27–29^. This is clearly shown by the model dynamics in Fig. 4. In this scenario, while a quite large number of people have a breakthrough infection (given a relatively low effectiveness of the vaccine against delta variant) their immune system is boosted and they stay immune for a longer period of time. Therefore, we expect a larger amount of cases in the short term, while in the long term, more people will remain immune, consistently reducing the number of cases over time. In this scenario, if the majority of the population loses their immunity, a new wave is expected in July 2022. This suggests the need of new vaccines, effective against new variants, in the years to come. Additionally, breakthrough infections could help reducing the number of cases in the long term while not increasing the amount of deaths.

The third scenario is depicted by the violet solid line in the figure. A smaller amount of cases is expected in the near future since most people are fully immunized. Afterwards, however, we expect a new growth of daily cases by the spring 2022. This growth is due to the loss of most people’s immunity. It is interesting to notice that, under our assumptions, the last peak in the 3rd scenario is predicted to be higher than in the 2nd one. This occurs because a lower efficacy of the vaccine implies a greater number of infections and, thus, re-immunized people. This antibody boost reduces the number of susceptible people by July 2022 lowering the height of the cases peak.

More data are still needed in order to decide which scenario is closer to the real one. However, our predictions raise questions about the proper management of the pandemic. How should vaccines be administered? Are non-pharmaceutical interventions a long-term solution? Is the pandemic here to stay? Our results suggest that new COVID-19 waves will come if highly transmissible, vaccine-resistant variants, are present. This would imply the need of new shots including strain-specific proteins. However, mass vaccination is clearly the key to reduce the appearance of new variants of concern and, consequently, the need of those specific shots. There is currently a huge debate in the world on this matter^30–32^. If vaccinated people get a mild version of the infection, COVID-19 will turn out to be a common, relatively harmless, influenza-like disease and (as shown in our model) there will be less susceptible people over time. Under these assumptions, mobility restrictions should be applied mainly to contain the spread of new variants of concern and boosters should only be given to high risk groups in the near future. Long-term booster vaccination is a different discussion to be considered, as there is no information on the duration of its immunity yet. The results of the present study seem to imply that the world’s population would probably need to be vaccinated periodically in the years to come. (See the Supplementary Materials for additional results.

In a changing scenario, during the publication process of this manuscript, the Omicron variant emerged, having a higher transmission coefficient, and becoming the prevalent variant worldwide. Updated results including this strain can be found in the Supplementary Materials.

### Discussion

Given the global impact of the COVID-19 pandemic, reliable models to analyze the virus propagation, including the variants of concern, are crucial to explore effective mitigation strategies. Our geo-stochastic multi-variant SEIRS-V model provides means of accurately describing the dynamics of the pandemic, as shown here. The model clearly separates the biological and social parameters. This property enables it to explain some global features observed in the daily number of worldwide cases. For instance, the surge of periodical waves is not strictly related to the appearance of new variants.These unexpected periods, have been seen in various countries with varying vaccination and social-distancing schemes. Our model can explain this global pattern, as naturally related to the biological parameters it includes, such as the immunity time of the recovered patients and the immunity time conferred by vaccines.

In the present work, we applied the model to the UK, and found that it successfully describes the dynamics of the pandemic. Notice that it was essential to fit variant-dependant epidemiological parameters like the transmission coefficients ($\beta _{k}$) for the Alpha and Delta variants of the disease. We identified the epidemiological parameters which determine the dominant variant: the one possessing larger transmission coefficient ($\beta$) or smaller exposed period ($\epsilon$) will eventually become dominant. Interestingly, as new variants with higher transmission coefficient appear, they quickly become the prevalent strain.

We emphasise that the model is applicable to any country with reliable data, as previously shown for Finland, Iceland, Mexico, Spain and Argentina^17–19^. Here we included the main variants of concern for the UK. The social parameters included in the model, as the different mobility types, allow to distinguish the effects of behavioural and cultural differences. Notice that in our model the biological parameters describe the properties of the disease, thus the same parameters could be used for any country, as in particular for Spain and Argentina is the case (see Supplementary Materials).

Furthermore, this model is useful to predict future scenarios, testing pharmaceutical and non-pharmaceutical interventions, and in particular to optimize the timing of vaccination boosters in order to minimize the appearance of new waves of the disease.

## Supplementary Information

Supplementary Information.

## References

[1] Dong E, Du H, Gardner L (2020). An interactive web-based dashboard to track COVID-19 in real time. The Lancet Infect. Dis..

[2] Ritchie, H. *et al.* Coronavirus pandemic (COVID-19). *Our World in Data* (2020). https://ourworldindata.org/coronavirus.

[3] World Health Organization. The effects of virus variants on COVID-19 vaccines (2021). https://www.who.int/news-room/feature-stories/detail/the-effects-of-virus-variants-on-covid-19-vaccines.

[4] COVID-19 Genomics UK Consortium. COG-UK / mutation explorer (2021). http://sars2.cvr.gla.ac.uk/cog-uk/.

[5] Centers for disease Control and Prevention. What you need to know about variants (2021). https://www.cdc.gov/coronavirus/2019-ncov/variants/variant.html.

[6] Burki T (2021). Understanding variants of SARS-CoV-2. The Lancet WORLD REPORT.

[7] Challen R (2021). Risk of mortality in patients infected with SARS-CoV-2 variant of concern 202012/1: matched cohort study. BMJ.

[8] World Health Organization. Tracking SARS-CoV-2 variants (2021). https://www.who.int/en/activities/ tracking-SARS-CoV-2-variants/.

[9] Collier, D. A. *et al.* Sensitivity of SARS-CoV-2 B.1.1.7 to mrna vaccine-elicited antibodies. *Nature* **293**, 136–141, 10.1038/s41586-021-03412-7 (2021). 10.1038/s41586-021-03412-7 PMC7616976 33706364

[10] Tillett RL (2021). Genomic evidence for reinfection with SARS-CoV-2: a case study. The Lancet Infect. Dis..

[11] Pedro, N. *et al.* Dynamics of a dual SARS-CoV-2 lineage co-infection on a prolonged viral shedding COVID-19 case: Insights into clinical severity and disease duration. *Microorganisms* **9**, 10.3390/microorganisms9020300 (2021). 10.3390/microorganisms9020300 PMC7912897 33540596

[12] Fontanet A (2021). SARS-CoV-2 variants and ending the COVID-19 pandemic. The Lancet, COMMENT.

[13] Ramos A, Vela-Pérez M, Ferrández M, Kubik A, Ivorra B (2021). Modeling the impact of SARS-CoV-2 variants and vaccines on the spread of COVID-19. Communications in Nonlinear Science and Numerical Simulation.

[14] Okabe Y, Shudo A (2022). Spread of variants of epidemic disease based on the microscopic numerical simulations on networks. Scientific Reports.

[15] Layton AT, Sadria M (2022). Understanding the dynamics of SARS-CoV-2 variants of concern in ontario, canada: a modeling study. Scientific Reports.

[16] Barrio, R. A., Varea, C., Govezensky, T. & José, M. V. Modelling the geographical spread of the influenza pandemic a(h1n1): The case of mexico. *Appl. Math. Sci* **7**, 2143–2176, 10.12988/ams.2013.13193 (2013).

[17] Barrio RA, Kaski KK, Haraldsson GG, Aspelund T, Govezensky T (2021). A model for social spreading of COVID-19: the case of mexico, finland and iceland. Physica A.

[18] Barreiro NL, Govezensky T, Bolcatto PG, Barrio RA (2021). Detecting infected asymptomatic cases in a stochastic model for spread of Covid-19. The case of Argentina. Scientific Reports.

[19] Barreiro NL (2022). Strategies for COVID-19 vaccination under a shortage scenario: a geo-stochastic modelling approach. Scientific Reports.

[20] WorldPop. Global high resolution population denominators project. *Funded by The Bill and Melinda Gates Foundation (OPP1134076) School of Geography and Environmental Science, University of Southampton; Department of Geography and Geosciences, University of Louisville; Departement de Geographie, Universite de Namur) and Center for International Earth Science Information Network (CIESIN), Columbia University*10.5258/SOTON/WP00670 (2018).

[21] Barreiro, N. L. Uk-map-code: First release v1.0, 10.5281/zenodo.6780779 (2022).

[22] Hodcroft, E. B. Covariants: SARS-CoV-2 mutations and variants of interest (2021). https://covariants.org/.

[23] Elbe S, Buckland-Merrett G (2017). Data, disease and diplomacy: Gisaid’s innovative contribution to global health. Global Challenges.

[24] Our World in Data. Coronavirus (COVID-19) vaccinations (2020). https://ourworldindata.org/covid-vaccinations.

[25] Thomas H (2021). A global panel database of pandemic policies (Oxford COVID-19 Government Response Tracker). Nature Human Behaviour.

[26] Pouwels, K. B. *et al.* Impact of delta on viral burden and vaccine effectiveness against new SARS-CoV-2 infections in the UK. *medRxiv*10.1101/2021.08.18.21262237 (2021). 10.1038/s41591-021-01548-7 PMC8674129 34650248

[27] Manisty C (2021). Antibody response to first BNT162b2 dose in previously SARS-CoV-2-infected individuals. The Lancet.

[28] Gobbi, F. *et al.* Antibody response to the BNT162b2 mRNA COVID-19 vaccine in subjects with prior SARS-CoV-2 infection. *Viruses* **13**, 10.3390/v13030422 (2021). 10.3390/v13030422 PMC8001674 33807957

[29] Goel, R. R. *et al.* Distinct antibody and memory B cell responses in SARS-CoV-2 naïve and recovered individuals after mRNA vaccination. *Science Immunology* **6**, 10.1126/sciimmunol.abi6950 (2021). 10.1126/sciimmunol.abi6950 PMC8158969 33858945

[30] World Health Organization. Interim statement on COVID-19 vaccine booster doses (2021). https://www.who.int/news/item/10-08-2021-interim-statement-on-covid-19-vaccine-booster-doses.

[31] Krause, P. R. *et al.* Considerations in boosting COVID-19 vaccine immune responses. *The Lancet, viewpoint*10.1016/S0140-6736(21)02046-8 (2021). 10.1016/S0140-6736(21)02046-8 PMC8437678 34534516

[32] Callaway E (2021). COVID vaccine boosters: the most important questions. Nature.

