# Supplementary material for: Modelling the interplay of SARS-CoV-2 variants in the United Kingdom
Source: Sci Rep. 2022 Jul 20;12:12372. doi: 10.1038/s41598-022-16147-w (PMC9296900; doi:10.1038/s41598-022-16147-w)
Supplement: Supplementary file 1 — Supplementary Information. [file 41598_2022_16147_MOESM1_ESM.pdf]

Supplementary Information on: Modelling the interplay of SARS-CoV-2 variants in the United Kingdom.

N. L. Barreiro, T. Govezensky, C. I. Ventura, M. N u nez, P. G. Bolcatto and R. A. Barrio

Model

Local dynamics. New compartments must be added to the SEIRS-V model, there are k E (exposed) and k I (infective) compartments, one compartment for each virus variant. S (susceptible), R (recovered) and V (vaccinated) are however, single compartments. Summation terms are used in R and S in order to include the individuals recovered after being infected with any of the variants, that later become susceptible again. Latency periods (ε_k), infectiousness periods (σ_k) and immunity periods (ω_k) are dimensionless constants expressed in units of one day.

V is a single compartment since available vaccines were all developed against the same variant. People become part of the vaccinated compartment only after receiving the shots recommended by pharmaceutics and having developed immunity. When vaccinated individuals get infected (with probability γ_k), the immune system is boosted by the presence of the virus, we therefore assume they will remain immune δ additional days after they got infected. Products in this V compartment track the timing of losing immunity of these individuals.

The dynamical model equations for each cell are described by the following mathematical map,

$$
\begin{aligned}
S(t+1) =& \mu + (1-\mu)\Bigg[S(t) - S(t)\sum_{k=1}^{N_{variants}} G_k(t) + \sum_{k=1}^{N_{variants}} z_k (1-\mu)^{\varepsilon_k+\sigma_k+\omega_k}\, S(t-\varepsilon_k-\sigma_k-\omega_k)\, G_k(t-\varepsilon_k-\sigma_k) - v_r(t)\, S(t) + \\
& (1-\mu)^{\delta}\, v_r(t-\delta)\, S(t-\delta) \prod_{\tau=t-\delta+1}^{t} (1-\sum_{k=1}^{N_{variants}} \gamma_k\, G_k(\tau)) + (1-\mu)^{\delta} V(t-\delta) \sum_{k=1}^{N_{variants}} \gamma_k\, G_k(t-\delta)\Bigg] \\
E_k(t+1) =& (1-\mu)\Bigg[E_k(t) + S(t) G_k(t) - (1-\mu)^{\varepsilon_k}\, S(t-\varepsilon_k)\, G_k(t-\varepsilon_k) + V(t)\, \gamma_k\, G_k(t) - (1-\mu)^{\varepsilon_k} V(t-\varepsilon_k)\, \gamma_k\, G_k(t-\varepsilon_k)\Bigg] \\
I_k(t+1) =& (1-\mu)\Bigg[I_k(t) + (1-\mu)^{\varepsilon_k}\, S(t-\varepsilon_k)\, G_k(t-\varepsilon_k) - (1-\mu)^{\varepsilon_k+\sigma_k}\, S(t-\varepsilon_k-\sigma_k)\, G_k(t-\varepsilon_k-\sigma_k) + (1-\mu)^{\varepsilon_k} V(t-\varepsilon_k) \\
& \gamma_k\, G_k(t-\varepsilon_k) - (1-\mu)^{\varepsilon_k+\sigma_k} V(t-\varepsilon_k-\sigma_k)\, \gamma_k\, G_k(t-\varepsilon_k-\sigma_k)\Bigg] \\
R(t+1) =& (1-\mu)\Bigg[R(t) + \sum_{k=1}^{N_{variants}} (1-\mu)^{\varepsilon_k+\sigma_k}\, S(t-\varepsilon_k-\sigma_k)\, G_k(t-\varepsilon_k-\sigma_k) - \sum_{k=1}^{N_{variants}} (1-\mu)^{\varepsilon_k+\sigma_k+\omega_k}\, S(t-\varepsilon_k-\sigma_k-\omega_k) \\
& G_k(t-\varepsilon_k-\sigma_k-\omega_k) + V(t-\varepsilon_k-\sigma_k) \sum_{k=1}^{N_{variants}} \gamma_k\, G_k(t-\varepsilon_k-\sigma_k) - (1-\mu)^{\delta} V(t-\delta) \sum_{k=1}^{N_{variants}} \gamma_k\, G_k(t-\delta)\Bigg] \\
V(t+1) =& (1-\mu)\Bigg[V(t) + v_r(t)\, S(t) - V(t) \sum_{k=1}^{N_{variants}} \gamma_k\, G_k(t) - (1-\mu)^{\delta}\, v_r(t-\delta)\, S(t-\delta) \prod_{\tau=t-\delta+1}^{t} (1-\sum_{k=1}^{N_{variants}} \gamma_k\, G_k(\tau))\Bigg]
\end{aligned}
\tag{1}
$$

$$G_k(t) = 1 - exp(-\beta_k\, I_k(t)) \tag{2}$$

Along the simulation, population is considered to be constant, $N = S + \sum_{k=1}^{N_{variants}} E_k + \sum_{k=1}^{N_{variants}} I_k + R + V$; if L denotes life expectancy, and for the mortality we assume an exponential functional form with a constant rate $\mu = 1/L$, birth rate should be μN; in each cell total population is normalized to 1. G_k is the Poisson based probability of becoming infected after being in contact with one or more infected people; the incidence function for each cell is $S(i,j,t)\rho(i,j)G_k(i.j.t)$, where β_k is the transmission parameter of each variant, it is a dimensionless constant which does not depend on population density or mobility of the population. Notice that in this SEIRS-V model, all time parameters remain constant, they depend only on each

virus variant and immune system's reaction, and are clearly separated from parameters affecting infection's spread. Actual population density within each cell is considered in the incidence function as indicated before; mobility parameters are involved in geographical spread, they are modulated by social compliance of non-pharmaceutical measurements proposed by different governments.

At local level, people do not only follow a daily routine but they may also visit unpredictable places. A local mobility parameter $0 < \nu_L < 1$ represents this fact, adding randomness to cell dynamics. ν_L is compared with a random number (r) from a uniform distribution: if $\nu_L \leq r$ the epidemics in the cell proceeds, otherwise no new cases are added in this cell this particular day.

Global dynamics. Traditional models use diffusion to model geographical spread of epidemics; we use a Metropolis Monte-Carlo algorithm to simulate mobility between cells. Although we described three different mobility parameters (ν_L, ν_n and ν_a), for simplicity we consider $\nu_L = \nu_n = \nu_a = \nu$, and $0 < \nu < 1$. In the case of neighbor cells, we localize cells where $I_k(i,j,t) \geq \eta$, then if $\nu \leq r$, the infections spreads to the neighboring cell (i,j+1,t) where $S(i,j+1,t)$ becomes $1-\eta$ and $I_k(i,j+1,t) = \eta$; (r is a random number from a flat distribution, η is a parameter related to the infectiousness of the disease).

For long distance dispersal, the amount of travels affects the probability of spreading the disease; to estimate this movement we consider that people travel more from/to large cities than from/to small ones, therefore, for long distance transmission from cell (i,j) where $I_k(i,j,t) \geq \eta$ to cell (m,n), if $\rho(i,j)\rho(m,n)\nu \leq r$ then $S(m,n,t) = 1-\eta$ and $I_k(m,n,t) = \eta$.

Non routinarytrips are captured in the model by the "kinetic energy" parameter kT. For cells with $\rho(i,j) > T$ (where T is a normalized threshold), if $exp(-kT) > r$ then $S(i,j,t) = 1-\eta$ and $I_k(i,j,t) = \eta$ meaning that an ill person reached this cell and propagated the disease.

Additional Results

Figure S1 shows the model applied to UK including the Omicron variant. From the simulations shown in the main text it is clear that scenario 1, as described in the main text, was the most similar to what actually happened, predicting a high peak of cases in UK at the beginning of 2022. The height and shape of the peak was modified by the surge of a new variant (the Omicron) and the government policies. In order to fit this new strain we used $\beta_{Omicron} = 11$, $\gamma_{Omicron} = 0.2$ and short vaccine immunity period ($\delta = 180$ days). In this case, booster vaccines were added to better model the evolution of the pandemic, as can be seen in figure S1(C) . Omicron has a very high transmission rate and prevailed over the other variants. It is expected that new variants will appear in the future.

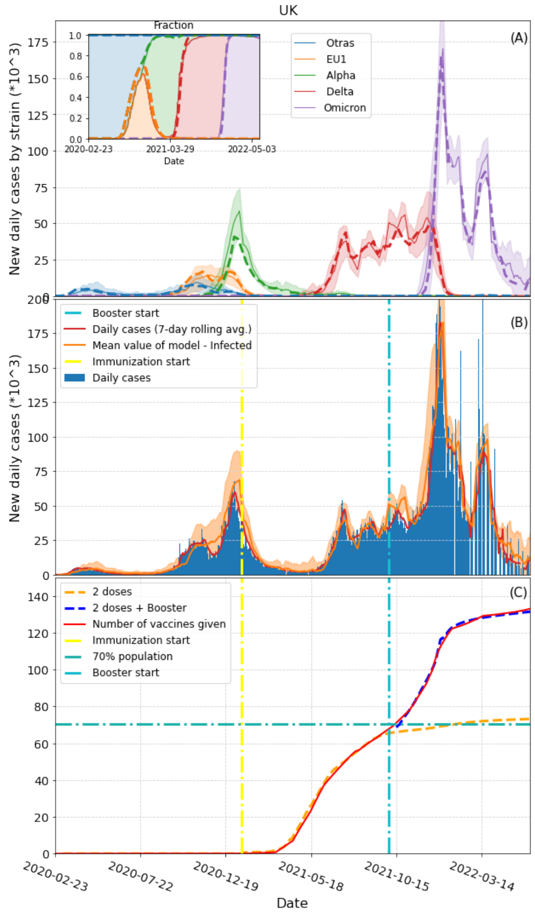

Figure S1. Model Applied to UK. γ_{Delta} was fixed to 0.01 (very high vaccine efficiency against the Delta variant, with immunity period of 180 days). The Omicron variant was fitted with $\beta_{Omicron} = 11$ and $\gamma_{Omicron} = 0.2$ All the other epidemiological parameters were kept as in Table 1 (main text). (A) Evolution of each variant over time. (B) Daily cases. (C) Percentage of immunized people. Notice that boosters were also included.

Figures S2 and S3 show the model applied to Argentina and Spain (without the Omicron variant). In both cases, the same biological parameters as in UK were used (see Table 1 in main text), $\delta = 180$ days and $\gamma_k = 0.01$, obtaining a good fit for all the variants. Notice that in our model the biological parameters describe the properties of the disease, thus the same parameters could be used for any country. In the case of Argentina we fitted β_k for the Gamma and Lambda strains with values 2.075 and 1.65, respectively. In the case of Spain we noticed that variant distribution is strongly dependent on geographical spreading. If a certain area is isolated from the others, waves of new strains won't surge there. Because of this, less transmissible variants may survive for longer periods of time. Since our model uses only one mobility parameter for the whole country, differences in social behaviour could not be captured. Particularly, the peak in December 2020 is formed by the contribution of 3 strains (Alpha, EU1, Other). One could assume that strains with higher transmission parameter (EU1, Alpha) will prevail, and the model predicts this behaviour as expected. However, particular geographical isolation makes the "other" variant to stay for a longer period. In this sense the model fails to capture the exact fraction of each variant in that specific peak.

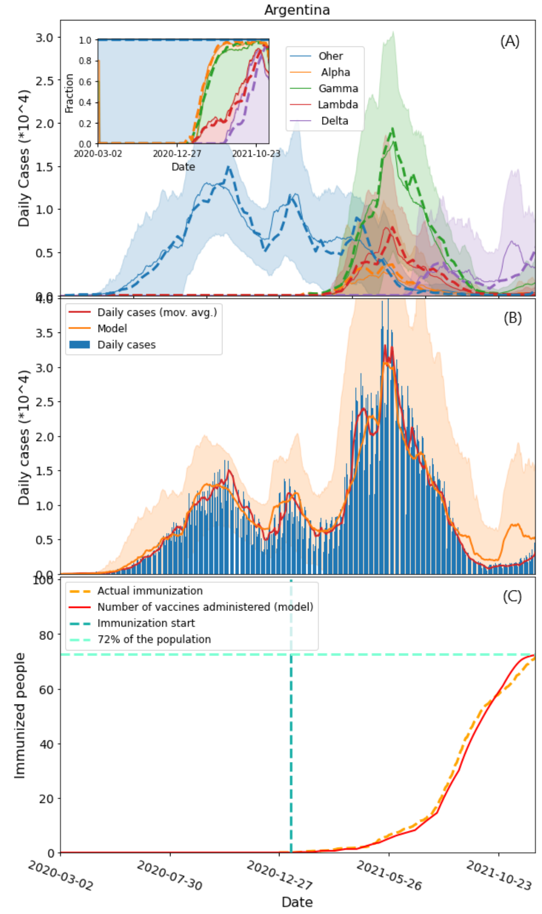

Figure S2. Model Applied to Argentina. Transmission parameters (β_k) are the same as in the UK for Alpha, Delta and other variants. β_k was fitted for the Gamma and Lambda strains with values 2.075 and 1.65, respectively. γ_k for Delta variant was fixed to 0.01 (very highs vaccine efficiency with immunity period of 180 days). All the other epidemiological parameters were kept as in Table 1 (main text). (A) Evolution of each variant over time. (B) Daily cases. (C) Percentage of immunized people.

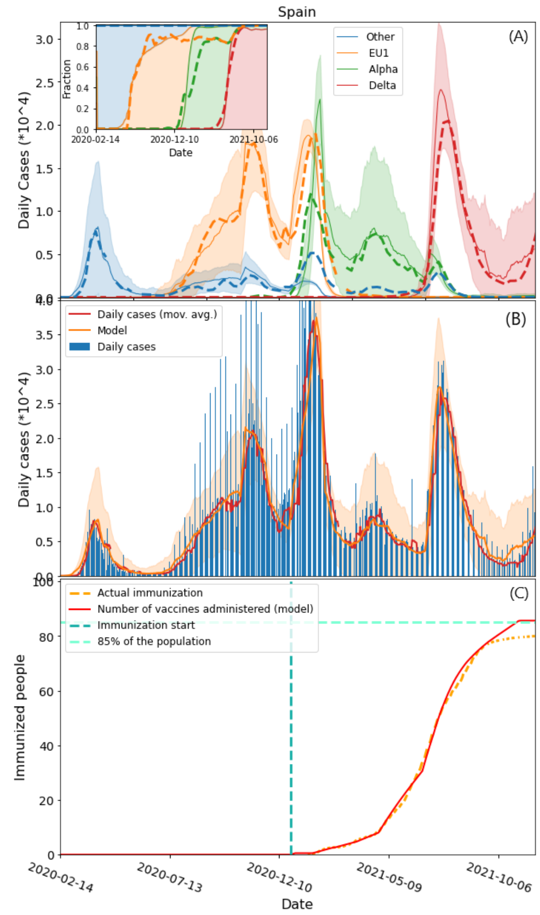

Figure S3. Model Applied to Spain. The transmission parameters (β_k) are the same as in UK for: EU1, Alpha, Delta and other variants. γ_k for Delta variant was fixed to 0.01 (very efficient vaccine, with immunity period of 180 days). All the other epidemiological parameters were kept as in Table 1 (main text). (A) Evolution of each variant over time. (B) Daily cases. (C) Percentage of immunized people.
